# Behavior, Process, and Evolution in the Multiscale Molar Paradigm

**DOI:** 10.1007/s40614-026-00501-8

**Published:** 2026-03-25

**Authors:** William M. Baum

**Affiliations:** 1 Department of Environmental Science and Policy, University of California, Davis, CA USA; 2 Walnut Creek, USA

**Keywords:** Molar view, Induction, Allocation, Covariance, Feedback system

## Abstract

The Multiscale Molar View of behavior fits with evolutionary theory and with General Process Theory. These two theories combine to afford a monistic ontological basis for understanding behavior as consisting of extended activities controlled by extended behavior–environment relations. General Process Theory casts the world as process and all existences as processes—no objects, no events, and no substance. Although the theory applies to both animate and inanimate things, behavior analysts focus on animate processes. Evolutionary theory conceives of a species as a process, the function of which is to evolve, and the individual members—the parts—as processes, the function of which is to reproduce. All behavior consists of activities, which are processes that ultimately promote surviving and reproducing. Evolutionary theory, process ontology, and the concept of induction provide a powerful conceptual framework for understanding not only behavioral phenomena in the laboratory and everyday life but also for more flexible approaches to clinical applications.

This article aims to outline briefly how a molar view of behavior fits with evolutionary theory and General Process Theory (Seibt, 2009), the philosophical framework that conceives the world to consist entirely of processes, which are temporally extended changes with beginnings and endings, rather than objects, events, or substances. Although General Process Theory expresses this conception in modern Western terms, it has many ancient precursors. In Hinduism, for example, it is expressed in the three principles symbolized by Brahmá, Vishnu, and Shiva (creator, sustainer, and destroyer; Meher Baba, 1973, p. 118). It is expressed also in truisms like “nothing lasts forever” and “the only constant is change.”

Baum (2018a, 2018b) proposed a multiscale molar view of behavior based on three laws: (1) the Law of Allocation, which states that competition among activities determines the allocation of time among an organism’s activities; (2) the Law of Induction, which states that events important to survival and reproduction (phylogenetically important events) induce specific activities; and (3) the Law of Covariance, which states that environmental feedback determines which stimuli become inducers and which activities are induced.

Evolutionary theory and General Process Theory support the framework expressed in the multiscale molar view. General Process Theory casts activities as processes for the Law of Allocation (Baum, 2018b). Evolutionary theory provides a conceptual framework for understanding the Law of Induction and the effects of inducers (Baum, 2024). Both evolutionary theory and General Process Theory illuminate the Law of Covariance, which applies to behavioral plasticity or adaptation. We begin with General Process Theory, then take up the concept of induction, and then the concept of covariance.

## General Process Theory

A process is, first, a change through time. It may involve locomotion, but not necessarily, as in processes of decay and deterioration. Second, a process has a beginning and an end. Every process comes into existence, lasts for a while, and then goes out of existence. It is possible that change and movement inspire the common constructs of time and space. Time may arise because processes have a beginning and end, which is labeled an amount of time, a duration. At any midway point in a process, some part seems past and some part yet to come. Space may arise when a process entails movement, which is labeled travel through space. Using these constructs, we may imagine a process to exist in four-dimensional space–time and to be like a thread in a tapestry composed of such threads in four dimensions. Third, processes have parts that are themselves processes. The parts of some processes are identical to the process of which they are part—for example, a part of an activity like watching television is some lesser duration of watching television. Parts of other processes are disparate processes that work together for the functioning of the whole process—for example, the parts of driving to work, which include processes like driving on a highway and stopping at red lights (Wallace, 1965). Seibt (2017) calls the former “automereous” and the latter “homomereous.”

According to General Process Theory, the world is one big process and only processes exist, not objects, not particulate events, and not even substance (see “The Myth of Substance,” Seibt, 2018). Talking about processes in English and other Indo-European languages is difficult, because these languages assume an ontology based on objects and discrete events (called by philosophers “continuants” and “occurrents”). The requirement that verbs have noun subjects leads to the inference of agency, which is inimical to science in general and a science of behavior in particular (Baum, 1995b). For example, we no longer think that “The sun rose above the horizon” implies that the sun or Apollo did anything, but according to folk psychology, “John rose from his chair” belongs in a different category, because John *did* the rising. Yet, for a science, both locutions just denote happenings (processes). Some non-Indo-European languages would allow talking about processes more easily. Seibt (2015) points to examples of languages with nuanced nouns, like Burmese, that contrast with the strict nouns of English. The linguist Whorf (1956) cites the examples of some American Indian languages that have no nouns, only verbs. Both Seibt and Whorf cite the impossibility of subject-less verbs in English, leading to constructions like “It is raining,” where “it” is a sort of dummy subject. We are stuck with English, however, and I will do the best I can with it.

A process is an occurrence or “happening” in space and time that has a beginning and an end and seen as temporally extended in time (“It’s snowing in Detroit,” “My chickens are laying eggs,” “I was studying for a test.”) Some are repetitive (chickens laying eggs) and some are continuous (snowing in Detroit). All processes, animate (e.g., organisms) or inanimate (e.g., houses, mountains, and rivers), have a beginning and an end. Everything changes, and nothing lasts forever. Animate processes differ from inanimate processes in one important way: animate processes resist their demise. Although General Process Theory deals with both animate and inanimate things, behavior scientists focus on living organisms and their behavior.

## Evolution and Process

Process ontology casts a species as a process (Ghiselin, 1997). The function of a species is to evolve. The members of the species, organisms, also are processes. The members are the parts that together make up the species (the whole). The function of an organism is to reproduce, and the reproducing of the members—differential reproducing among members—results in evolution of the species (Nicholson & Dupré, 2018).

How did these phenomena come about? Organisms exist because sequestering genetic material (DNA, the “replicator”) inside an “interactor” results in more copies and more faithful copies of the DNA (Hull et al., 2001). These advantages selected the genes that make organisms in the competition with less-organized life forms, such as bacteria and viruses, a competition that still persists today, as our immune system fights off infections. Organisms exist to reproduce, although many members of a population might produce few offspring or none at all. The population remains stable as long as enough members produce enough offspring. Whatever other processes may occur in an organism, like staying alive, the ultimate function of an organism is reproducing. Staying alive usually serves reproducing, because organisms can only reproduce if they stay alive long enough to reproduce. Exceptions prove the rule, as when parents sacrifice themselves for the sake of their offspring.

Every process is a “nonparticular individual” (Baum, 2017b; Seibt, 2009). By “individual” is meant an integral, functioning whole with parts that together make it function. A species is an example of an individual (Ghiselin, 1997). “Nonparticular” means not momentary, extended in time. An organism is a nonparticular process. In speaking of behavior, we may say that an organism, as a process, has parts and processes that may be called *activities*. An activity is a nonparticular process of an organism that interacts with the surrounding environment (Baum, 2002, 2024). As a process, an activity has parts, also processes, that work together to realize the function of the activity (Baum, 2013). The parts of a tennis serve—placing feet, throwing the ball up, bringing down the racquet, etc.—work together to get the ball moving rapidly over the net.

Like any individual process, an organism comes into existence at some point in four-dimensional space–time, lasts for a while, and then ends or dies. A key characteristic of an organism is that it must be continually active if it is to stay alive. As a thermodynamic system, the organism must take in energy from the environment and expel waste into the environment continuously to keep its entropic state from increasing and moving toward the equilibrium that is death (Nicholson, 2018). The state it maintains away from equilibrium while it is alive is sometimes called “homeostasis.” The organism’s means of taking in energy from the environment, in the form of food and other resources, is what we mean by “behavior.” Physiological processes also contribute to maintaining the organism alive and may be distinguished from behavior in that they do not necessarily interact with the environment (e.g., the heart beating and hormonal effects), but the distinction is not always clear-cut (e.g., sweating).

Behavior that threatens the health and safety of oneself and others, when not sanctioned by society (e.g., fighting in a war), is often labeled as “maladaptive.” Maladaptive behavior typically results from conflict between short-term and long-term relations between behavioral alternatives and the environment. Short-term relations may exert strong control, and acting in accord with them, when maladaptive, is often called “impulsive.” Examples are squandering one’s earnings instead of saving or eating junk food instead of healthy items. Acting in accord with competing long-term relations is often called “self-control” (Rachlin, 2000).

Dysfunction also occurs when the environment changes rapidly. The climate becomes hotter or colder, wetter or dryer, addictive substances like sugar, alcohol, heroin, and cocaine become available in unnatural quantities, or waste products accumulate excessively. When that happens, physiology and behavior must adapt, or else organisms die and fail to reproduce, and species go extinct. In humans, adaptation often relies on cultural change, because genetic evolution will usually be too slow. Law enforcement and education change individual norms of behavior—i.e., rules and cultural activities, or practices (Baum, 1995a, 2000).

## Evolution and Induction

Certain events cause major changes in behavior when present in the environment. Events like encountering a potential mate, food, injury, or a predator induce specific activities just by their presence. The activities they induce vary, depending on the type of event. Food or a potential mate induce activities that make contact more likely, whereas injury or predators induce activities that make contact less likely. A reasonable explanation for such effects appeals to natural selection. Individuals in a population that fail to respond to such events are less likely to leave offspring, because they will gain fewer resources or be eaten. Thus, populations typically comprise individuals that respond vigorously to these events. For this reason, these inducing events may be called “Phylogenetically Important Events” (PIE), where “phylogenetically important” means causing natural or sexual selection. Borgstede and Eggert (2021) call them “statistical fitness predictors” to emphasize that they may affect fitness.

PIEs not only induce nonoperant activities by their presence, they also induce the operant activities that produce the PIEs. If lever pressing produces food, the food also induces the lever pressing. The increase and maintenance of operant activities that was historically called “reinforcement” in this framework is an example of *induction*, the concept introduced by Segal (1972). The behavior–environment feedback system thus consists of operant activity as output from the organism and inducing feedback from the environment. For example, when an operant activity like key pecking in a pigeon produces food according to a ratio or interval schedule, the food produced induces more of the operant activity and maintains it if a balance between production and induction is possible (Baum, 2025). Not only food, but also electric shocks induce lever pressing if pressing a lever avoids the shock (avoidance; Baum, 2020).

## Process and Covariance

The molar view of behavior takes behavior to consist of activities, which are inherently extended in time. It eschews the traditional notions of discrete response, contiguity of events, and strengthening by reinforcement—the *molecular* view. Instead, the molar approach sees behavior and the environment as in constant exchange with one another, as in a feedback system (Baum, 1989, 2025). The relations between activities and environmental provision of essentials necessary to survival and reproduction are called feedback functions. A ratio schedule, for example, may provide food in direct proportion to the activity that produces it—foraging for seeds or hunting clams, for example.

The law of covariance states that activities that covary with PIEs are selected because the PIEs induce these operant activities. Feedback functions describe covariance. They show how the flow of behavior results in a flow of PIEs (e.g., food).

**Fig. 1:**
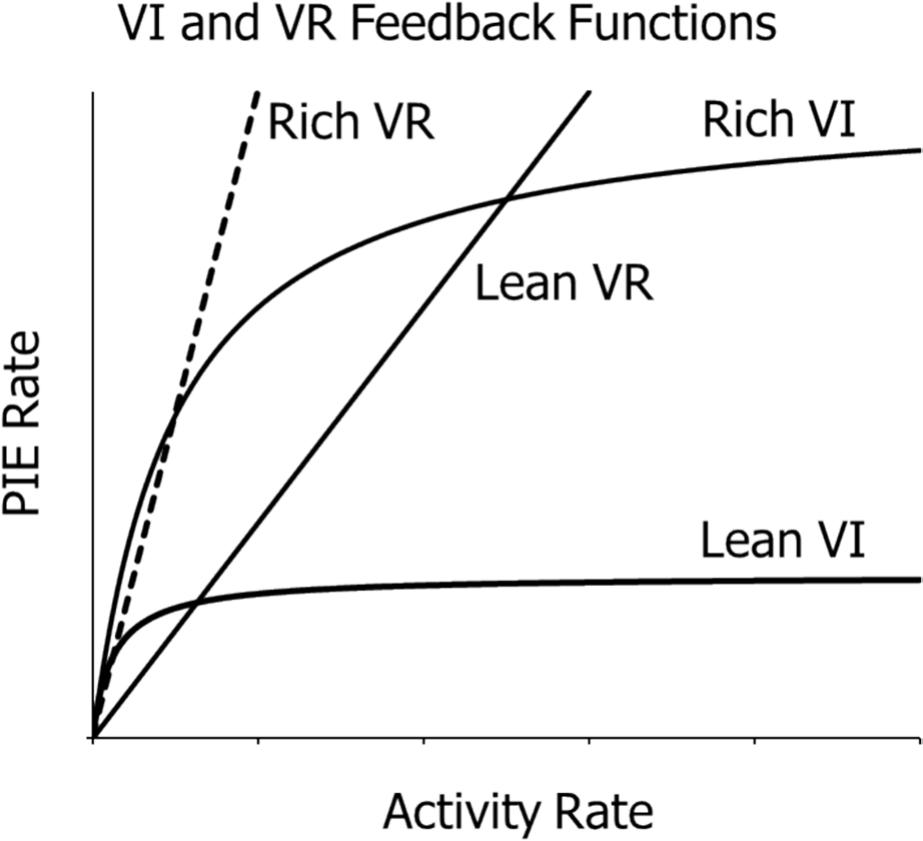
Examples of Feedback Functions for Ratio (VR) and Interval Schedules (VI). *Notes.* Straight lines describe functions for a lean and a rich ratio schedule. Curves describe functions for a lean and a rich interval schedule. The dashed line shows the upper limit to ratio schedules, where PIE rate equals activity rate

Figure 1 shows some examples. The straight lines describe feedback functions for two ratio schedules. The food rate is directly proportional to the activity rate. The ratio schedule determines the slope of the line and, thus, how rich or lean the schedule may be. The curves describe feedback functions for two interval schedules. The slope is steep at low activity rates and gradually approaches zero as the food rate approaches the programmed rate as an asymptote, which specifies how rich or lean the schedule may be. When these feedback functions are combined with Eqs. 1 and 2 (see below) in a feedback system, explanations and predictions of the performances emerge (Baum, 2025). The different feedback functions result in different performances.

Feedback functions are examples of covariance between an activity and the results it produces. They are flow relations extending through time, but they only exert control over behavior if they are tight and not too variable (Baum, 2022). They may be extracted from data (e.g., Baum, 2012a, Fig. 17; Baum & Grace, 2020, Fig. 7), and thus may induce cultural rules like “Eat a healthy diet,” when the covariance between eating a poor diet and long-term health may be obscure (Baum, 1995a, 2000). In the laboratory, they may be programmed with varying degrees of closeness or tightness (Baum, 2022). The effectiveness of a covariance often requires close grouping in time between activity and consequences, which entails proximity, but does not entail contiguity between discrete events in the traditional sense. Sometimes the relation need only be highly reliable, even if not close in time—e.g., the relation between storing food during abundance and eating during scarcity. Instead of the molecular concepts, the molar view proposes that behavior and environment may be likened to a feedback system (Baum, 1973, 1981, 1989, 2025).

The law of covariance also states that signals that covary with a PIE become proxies for the PIE. Such conditional inducers induce most of the same operant activities as the PIEs and explain stimulus control, classical conditioning, and conditioned reinforcement (Baum, 2012a). With signal-PIE covariance, the concept of induction unites all the disparate concepts of stimulus control: conditional stimuli of Pavlovian conditioning, discriminative stimuli of operant control, and conditioned reinforcers all are conditional inducers.

## Visualization

**Fig. 2:**
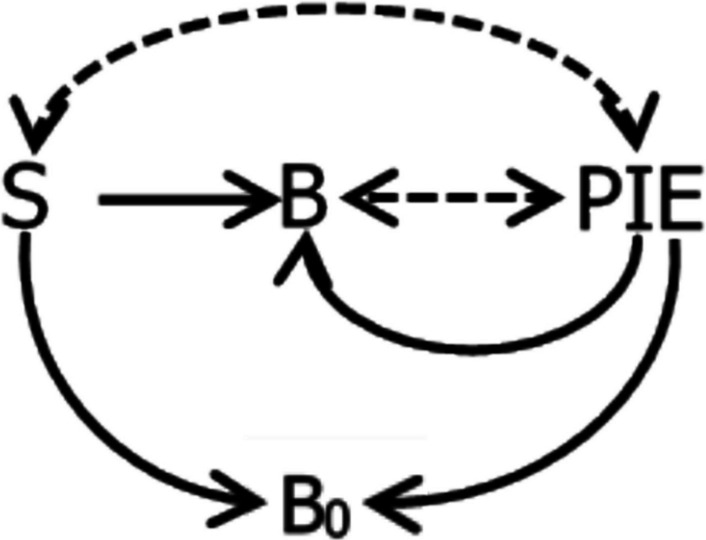
Graphical Representation of Covariance between Context (*S*) and a PIE (e.g., Food), Covariance between an Operant Activity (*B*) and the PIE, Induction of the Operant Activity by *S* and the PIE, and Induction of Nonoperant Activities (*B*_*0*_) by *S* and the PIE. *Note.* Solid arrows indicate induction. Dashed two-headed arrows indicate covariance

Figure 2 illustrates the basic concepts in this conceptual framework in graphical form. The two-headed dashed arrows indicate covariance, and the solid arrows indicate induction. The operant activity (*B*) produces the PIE and so is in covariance with the PIE (dashed arrow). The context or signal (*S*) is in covariance with the PIE (dashed arrow) as long as *B* is producing the PIE. Both the PIE and *S* induce the operant activity *B* (solid arrows). In addition, both the PIE and the context induce nonoperant activity (*B*_*0*_), which competes with the operant activity (Baum & Aparicio, 2020; Breland & Breland, 1961).

The relations shown in Fig. 2 diagram the situation conventionally known as “positive reinforcement.” The “reinforcer” is a PIE or PIE proxy that induces the operant activity (*B*). The increase and maintenance of the operant activity (*B*) is conventionally called “reinforcement.” The context *S* includes both present and past events (signals and history). Its effects on the operant activity *B* is conventionally termed “discriminative stimulus control.” The inducing of *B*_*0*_ is conventionally known as “Pavlovian” or “respondent.” This view of operant behavior requires no hypothetical constructs, such as “strength” (Simon et al., 2020).

The molecular view, with its discrete responses and immediate consequences, always had trouble explaining avoidance, because when a consequence is avoided, the activity is followed by nothing. Molecular theorists dealt with this inadequacy by inventing an inner “fear” that was reduced when the avoidance response occurred—so-called “two-factor theory,” which, though irrefutable also seemed implausible (Herrnstein, 1969).

The molar view has the strength of treating avoidance in a clear way and without hypothetical constructs like “fear” (Baum, 2020). Avoidance is just operant activity that postpones, prevents, or mitigates a PIE or PIE proxy that threatens or is contrary to survival and reproduction. Again context includes both present signals and past history. Again context and the PIE may induce activities (*B*_*0*_) that interfere with avoidance.

**Fig. 3:**
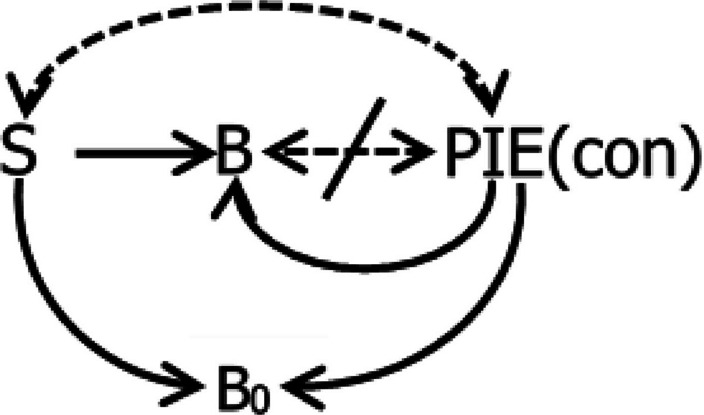
Graphical Representation of Covariance between Context (*S*) and a PIE Contrary to Survival and Reproduction (“con”; e.g., Shock), Inverse Covariance (Slash) between Operant Avoidance Activity (*B*) and the PIE, Induction of the Operant Avoidance Activity by *S* and the PIE, and Induction of Nonoperant Activities (*B*_*0*_) by *S* and the PIE. *Notes.* Solid arrows indicate induction. Dashed two-headed arrows indicate covariance

Figure 3 diagrams avoidance. It is the same as Fig. 2 except for two differences. First, the slash through the double arrow between the operant activity (B) and the PIE indicates the covariance is inverse—that is, the higher the rate of B, the lower the rate of the PIE, and vice versa. Second, the PIE is labeled “con” to indicate that it is contrary or threatening to survival and reproduction. Like Figs. 2 and 3 shows how the context *S* and the PIE induce *B*_*0*_, which may be activities like freezing and immobility interfering with the avoidance *B*.

Various situations may be seen as avoidance in the light of Fig. 3. In the laboratory, electric shocks induce lever pressing that postpones the shocks, and the activities that interfere with the lever pressing correspond to what Bolles (1970) called “species-specific defense reactions.” A recovered alcoholic refuses an alcoholic drink (*B*) and takes soda water, avoiding falling off the wagon (PIEcon). A person with a nut allergy examines grocery labels and avoids any items that might contain traces of nuts and trigger illness (PIEcon). Tom turns down invitations to social gatherings where his ex-wife will be present, avoiding hostile interactions (PIEcon). A child acts out in the classroom, avoiding assigned class work. Agoraphobia is likely avoidance, but the therapist must discover what PIEcon is being avoided.

## Three Laws of Behavior: Quantification

The three laws, applied with additional assumptions to specific situations, allow explanations of behavioral phenomena both in the laboratory and in everyday life (Baum, 2024). Together, the laws of allocation, induction, and covariance lead to quantitative accounts that allow mathematical predictions (Baum, 2018a, 2018b).

Whenever experimental conditions have allowed quantification, we find that the law of induction states that PIEs induce operant and nonoperant activities according to power functions—that is, the ability of an activity to compete with other activities, its *competitive weight V*, varies according to:

1 $$V=c{x}^{S}$$

where *x* is a variable like rate, amount, or immediacy of the PIEs, the coefficient *c* reconciles units, and the exponent *s* indicates the power of the inducing PIEs.

**Fig. 4:**
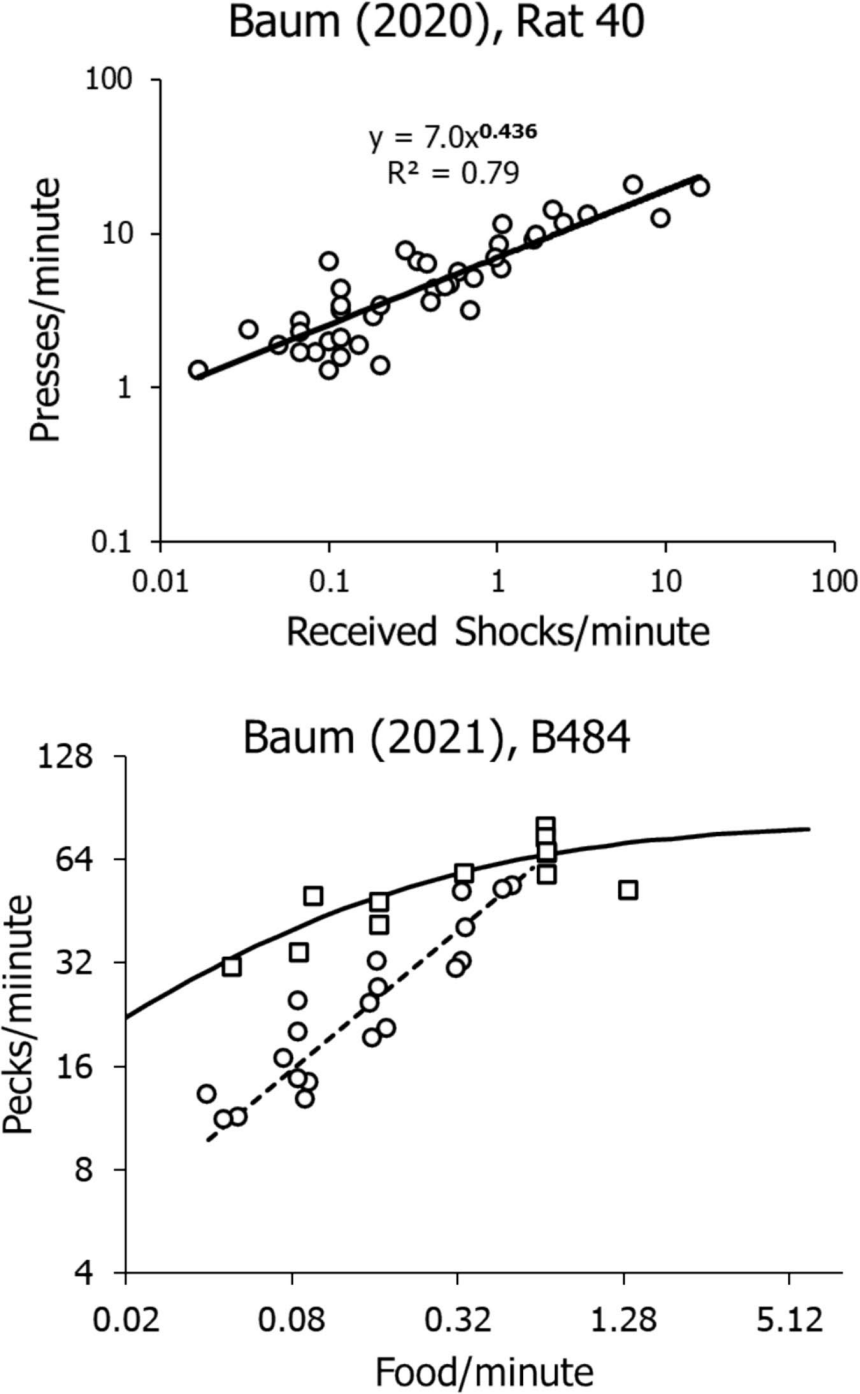
Two Examples of Power-Function Induction Observed in Experiments. *Notes*. **Top**: Reanalysis of Sidman’s (1962) avoidance data including a wide variety of conditions with different parameters. Note logarithmic coordinates. **Bottom**: Results from an experiment with pigeons. Circles show rates from a series of conditions in which overall food rate was held constant. The dashed line shows power-function induction. Squares show rates from a series of VI schedules. The solid curve represents the fit of Eq. 4. Note logarithmic coordinates

Figure 4 shows two examples of empirical demonstrations of Eq. 1. The top graph shows my reanalysis of one of Sidman’s (1962) data sets from his study of free-operant avoidance (more in Baum, 2020). In these logarithmic coordinates, the power function of Eq. 1 appears as a straight line.

Baum and Rachlin (1969) pointed out that time taken is a universal measure of behavior. Whatever else we may measure about an activity, such as the energy it consumes, we measure its frequency or prevalence by the amount of time the activity takes up. In everyday and clinical settings, an observer may record the time manually. In the laboratory, where experiments are automated, time is often estimated by attaching a switch to a device like a lever, key, or button and counting operations of the switch (Baum, 1975, 1976; Baum & Rachlin, 1969). Each switch operation corresponds to an amount of time interacting with the device. Baum (1976), for example, showed that counting rats’ lever presses resulted in the same choice relations as measuring the amount of time the lever was depressed. Dividing the number of switch operations by the total time available yields a “response rate,” so-called because in the molecular view each switch operation is a bit of behavior, a discrete response. In the molar view, because the number of switch operations measures the time taken by interacting with the device, the rate of switch operations measures the proportion of time interacting with the device.

The law of allocation states that relative competitive weight (Eq. 1) determines proportion of time an activity takes (Eq. 2). With their various competitive weights, an organism’s activities compete for time, because time is always limited. The law may be stated in the general equation:

2 $$\frac{{T}_{j}}{\sum_{i=1}^{N}{T}_{i}}=\frac{{V}_{j}}{\sum_{i=1}^{N}{V}_{i}}$$

where *T*_*i*_ is the time taken by Activity *i*, and *V*_*i*_ is the competitive weight of Activity *i* out of the *N* activities that occur. The equation states that the proportion of time taken by any one activity, out of the total time available ($\sum T$) equals (i.e., matches) the competitive weight of the activity relative to the total competitive weight of all the activities ($\sum V)$. If the competitive weight of any activity increases or decreases, other activities’ times also must decrease or increase. An increase in one means a decrease in others, and a decrease in one means an increase in others, because the activities together take up all the time. If I spend more time working, I may have to spend less time with family or sleeping, because a day contains only 24 h. Induced activities generally compete, but if an induced activity has homomereous parts, those parts may be seen to “collaborate” (Lopez-Tolsa & Pellon, 2025).

The simplest possible version of Eq. 2 applies to a situation with one operant activity. At least two other activities must be incorporated: other, nonoperant activities induced by the PIE (*T*_*0*_) and other activities unrelated to the PIE (*T*_*N*_). The equation takes the form:

3 $$\frac{{T}_{1}}{{T}_{1}+{T}_{0}+{T}_{N}}=\frac{{V}_{1}}{{V}_{1}+{V}_{0}+{V}_{N}}=\frac{{c}_{1}{x}^{{S}_{1}}}{{c}_{1}{x}^{{S}_{1}}+{c}_{0}{x}^{{S}_{0}}+{V}_{N}}$$

where *T*_*1*_ is the time taken by the operant activity. In the spare conditions of the laboratory, usually *V*_*N*_ may be considered negligible, because it usually has very low competitive weight relative to *V*_*1*_ and *V*_*0*_. In the everyday world, *V*_*N*_ could be large—say, in the competition between work and family. Podlesnik and Baum (2024) used Eq. 3 to model resurgence.

For research, Eq. 3 may be simplified with two assumptions. First, because the sum *T*_*1*_ + *T*_*0*_ + *T*_*N*_ equals the sum of all the time available, we set it equal to a constant *K*. Second, we assume *V*_*N*_ is negligible. This allows dividing the numerator and denominator in the expression on the right by ${c}_{1}{x}^{{s}_{1}}$ and we arrive at:

4 $${T}_{1}=\frac{K}{1+c{x}^{-s}}$$

where *c* is the ratio $\frac{{c}_{0}}{{c}_{1}}$ and *s* is the difference *s*_*1*_-*s*_*0*_. If *s*_*1*_ is greater than *s*_*0*_, Eq. 4 predicts that *T*_*1*_ increases with increasing *x* in a curvilinear pattern, approaching *K* as an asymptote (Fig. 3, bottom). If *s*_*0*_ were to exceed *s*_*1*_, *T*_*1*_ would decrease as *x* increased, an effect that has been called “misbehavior” (Breland & Breland, 1961). Equation 4 has been applied in a large number of analyses (e.g., Baum, 2021; Baum & Aparicio, 2020).

## Applied and Clinical Implications

Waltz and Follette (2009) identified four functional relations that stem from the molar view of behavior and would be potentially useful in therapeutic clinical applications. The first is matching theory. They present a version of Eq. 3 as a way to frame interventions that might decrease problem (target) activity (*T*_*1*_ here) and increase desirable behavior (*T*_*0*_). The implications for assessment point to the need to monitor alternative activities, not just the target activity. Interventions they suggest function by decreasing *V*_*1*_ or increasing *V*_*0*_, both of which increase the relative competitive weight of *T*_*0*_.

The second functional relation Waltz and Follette (2009) identify is the concept of discounting, which they characterize as: “how the value of a reinforcer is degraded when some form of inconvenience (e.g., delay, risk, or cost) accompanies it” (p. 56). These factors all may reduce the effectiveness of the “reinforcer” as an inducer. They might decrease either the exponent or the coefficient or both in Eq. 1. If so, they shift relative competitive weight toward the alternative that tends to produce powerful short-term inducers, the activity often labeled “impulsive.” Waltz and Follette cite a large body of research that has produced methods of assessing impulsive choosing of short-term inducers versus choosing of more favorable long-term inducers (“self-control”). They describe treatments that aim to help clients to favor the long-term relations.

Waltz and Follette (2009) call the third concept “behavioral momentum,” defined as “a behavior pattern’s resistance to change when faced with challenging situations” (p. 60). Persistence of desirable activities in the face of challenges and persistence of undesirable activities despite challenges may be addressed in a molar view of behavior without vague or hypothetical constructs like “strength.” Waltz and Follette discuss environmental variables like context and verbal rules that might be modified for treatment. Early research suggested that high reinforcer rate might increase momentum (e.g., Nevin, 1992), but subsequent experiments indicate that this effect is ephemeral when subjects are well-experienced (Baum, 2012b, 2021; Bell & Baum, 2021).

The fourth factor Waltz and Follette (2009) discuss is variability, a molar feature of behavior that can only be assessed over time. They point out that too little variability—stereotypy—may be undesirable, but also that too much variability may undermine desirable behavior patterns. They suggest methods for training both higher and lower variability.

The concept of induction shows up indirectly in applied and clinical research and practice in two ways. First, recognition of the “discriminative properties of reinforcers” acknowledges that, like so-called discriminative stimuli, “reinforcers” are inducers of activities, either unconditional (PIEs) or conditional (proxies). Experiments on choice with pigeons (Davison & Baum, 2006, 2010; Krageloh et al., 2005), rats (Bolles, 1961), and children (Cowie et al., 2021) have studied situations in which delivery of a “reinforcer” signals “reinforcer” availability for an alternative other than the just-productive alternative. These experiments have shown that when the signaling function of “reinforcers” is separated from the putative “strengthening” function, the signaling (i.e., inducing) function prevails. In a different sort of procedure, Wood and Simon (2021, 2023), working with normally developing and autistic children, found that the time taken to change from one activity to another depended on the inducers available in the prospective activity, rather than the inducers available in the previous activity. Instead of assuming that “reinforcers” strengthen behavior, applied behavior analysts might consider such signaling or inducing functions when designing interventions.

The second way induction shows up is in the application of “noncontingent reinforcement,” a phrase that is an oxymoron, because the definition of “reinforcement” includes its being contingent. Instead, the procedure may be seen as the presentation of inducers. Their usefulness arises because the “noncontingent reinforcers” induce (nonoperant) activities that compete with other activities, particularly undesirable activities.

## Conclusion

This conceptual framework applies to laboratory studies and also to everyday life—whether to experiments on choice (Baum, 1974, 1979) or to why a person would work for wages (Baum, 2017a). Clinicians too may find this conceptual framework more useful than traditional reinforcement theory. The Multiscale Molar View (Baum, 2018a, 2018b) encompasses the concept of reinforcement as an instance of induction. In addition, it offers a mechanism for stimulus control (also induction) and a way of understanding avoidance (induction of avoiding by encounters with the thing avoided; Baum, 2020)—and the effects of punishment—induction of alternative activities that avoid the punisher (Baum, 2024). Working within this conceptual framework, applied behavior analysts need not contrive or imagine discrete responses or close temporal pairings, instead measuring activities and behavior–environmental relations as temporally extended and taking time (e.g., Wood & Simon, 2021, 2023). Moreover, the Multiscale Molar View points to the importance of nonoperant activities, which often compete with operant activities and may both interfere with treatments and suggest new approaches to treatment (Baum & Aparicio, 2020). The book, *Introduction to Behavior: An Evolutionary Perspective* (Baum, 2024), offers a grounding in this conceptual framework.

## Data Availability

All data in this article are published and publicly available.
